# Is set-shifting and central coherence in anorexia nervosa influenced by body mass index, anxiety or depression? A systematic review

**DOI:** 10.1186/s12888-021-03120-6

**Published:** 2021-03-08

**Authors:** Tone Seim Fuglset

**Affiliations:** Møre and Romsdal Hospital Trust, Molde Hospital, Parkvegen 84, 6412 Molde, Norway

**Keywords:** Anorexia nervosa, Set-shifting, Central coherence, Anxiety, Depression, Body mass index, Systematic review

## Abstract

**Background:**

Anorexia nervosa (AN) is a severe eating disorder, recognized by a relentless pursuit for thinness and extreme low body weight. The disorder is often accompanied by comorbid disorders such as anxiety and depression, and altered neuropsychological function in terms of poor set-shifting and reduced central coherence. The aim of this review was to evaluate whether neuropsychological impairments in AN are influenced by body mass index, anxiety or depression.

**Method:**

A systematic review approach was used, following the PRISMA guidelines for systematic reviews. Literature was identified via searches in PubMed, PsychInfo and Embase database, by using the search words [anorexia nervosa] AND [central coherence], and [anorexia nervosa] AND [set-shifting]. Studies were included if they were written in English, peer-reviewed, included individuals with AN, included tests measuring set-shifting and/or central coherence, investigated associations between set-shifting/central coherence with anxiety and/or depression and/or BMI. Risk of bias was assessed by using a critical appraisal checklist from the Joanna Briggs Institute. Results were summarized in a narrative synthesis.

**Results:**

Although results are heterogeneous, the majority of studies report that neither body mass index (BMI), anxiety or depression is associated with altered central coherence and set-shifting in individuals with AN.

**Conclusions:**

Findings indicate that BMI, depression and anxiety does not influence neuropsychological function in AN, suggesting that it could be a characteristic of the disorder. A complete understanding of predisposing, precipitating and maintaining factors in AN needs to be addressed in future research. This could contribute to the development of better and more targeted treatment strategies.

**Supplementary Information:**

The online version contains supplementary material available at 10.1186/s12888-021-03120-6.

## Background

Eating disorders are characterized by a persistent disturbance of eating or eating-related behaviour that result in altered consumption of food, which significantly impairs physical health or psychosocial functioning [1]. Anorexia nervosa (AN) is a severe eating disorder, with the highest mortality rate of any mental illness [2]. It is recognized by a relentless pursuit for thinness, extreme low body weight, and is often accompanied by a disturbed body image and a denial of illness. The aetiology of AN remains unclear, however the disorder is most likely caused by a complex interaction between genetic and environmental factors. Genetic variants significantly associated with AN have recently been identified, which demonstrates that the origins of this disorder is both metabolic and psychiatric [3].

Neuropsychological function in AN has been investigated in a number of studies, suggesting that specific neuropsychological impairments may be related to the pathophysiology of the disorder. These studies show that individuals with AN often display poor set-shifting abilities and weak central coherence (for systematic reviews see [4, 5]). Set-shifting, or cognitive flexibility, refers to the ability to shift thoughts or actions according to situational demands. Central coherence is the degree to focus on details in processing information and the global integration of such information [6]. Individuals with weak central coherence have a preference for details over global processing. These findings are most commonly seen in adults, and findings from studies including adolescents are less consistent [7, 8]. It is worth noting that adolescence is a time of considerable development of the brain and cognitive abilities, affecting the development of both executive functions and social cognition [9].

Neuropsychological impairments in AN could be a characteristic of the disorder, however, it could also be a consequence of malnutrition and underweight. Yet, the role of starvation is still unclear, as some neuropsychological functions remains intact and general intelligence is within the normal range [10]. In fact, studies have shown that patients with AN perform better on selected tasks, such as tasks requiring local information processing [5, 11, 12]. A review and meta-analysis state that individuals with AN score above the average intelligence quotient of the normative population in the National Adult Reading Test and Wechsler Intelligence Scales [13].

Individuals with AN often suffer from comorbid mental disorders. The most common comorbidities are mood disorders, personality disorders, anxiety disorders, obsessive-compulsive disorders, and developmental disorders (e.g. autism spectrum, attention-deficit hyperactivity disorder) [14]. Both depression and anxiety have been linked to cognitive impairments in the domains of episodic memory, verbal memory as well as varying subsets of executive function [15, 16]. However, the role of depression and anxiety on neuropsychological performance in individuals with AN remains unclear. On one hand, comorbid psychiatric symptoms such as depression and anxiety may contribute to poorer performance on neuropsychological tasks. On the other hand, poorer performance could also be a characteristic of the disorder, and unrelated to non-eating disorder psychopathology.

A complete understanding of neuropsychological function in AN may contribute to the development of better and more specific treatment strategies. It would be beneficial to determine whether neuropsychological impairments are related to comorbid disorders or as a consequence of underweight, or whether these are more likely a characteristic of the disorder per se. If so, this should be targeted in treatment.

Set-shifting and central coherence are the most studied cognitive domains in individuals with AN. This systematic review provide an overview of relevant studies and their findings concerning the relationship between clinical measures and neuropsychological function in AN. The aim of the current systematic review is to evaluate whether the observed alterations in set-shifting and central coherence in patients with AN are influenced by body mass index (BMI), ED severity and broader transdiagnostic factors such as anxiety and depression.

## Methods

This systematic review was guided by the PRISMA criteria [17].

### Eligibility criteria

All primary studies were peer-reviewed and written in English. There were no limitations in terms of time frame. Studies were included if they met the following inclusion criteria:

1. Manuscript written in English
2. Study published in peer-reviewed journals
3. Included individuals diagnosed with AN
4. Included tests measuring set-shifting and/or central coherence
5. Investigated associations between set-shifting/central coherence with anxiety and/or depression and/or BMI

### Search strategy and selection of studies

**Fig. 1:**
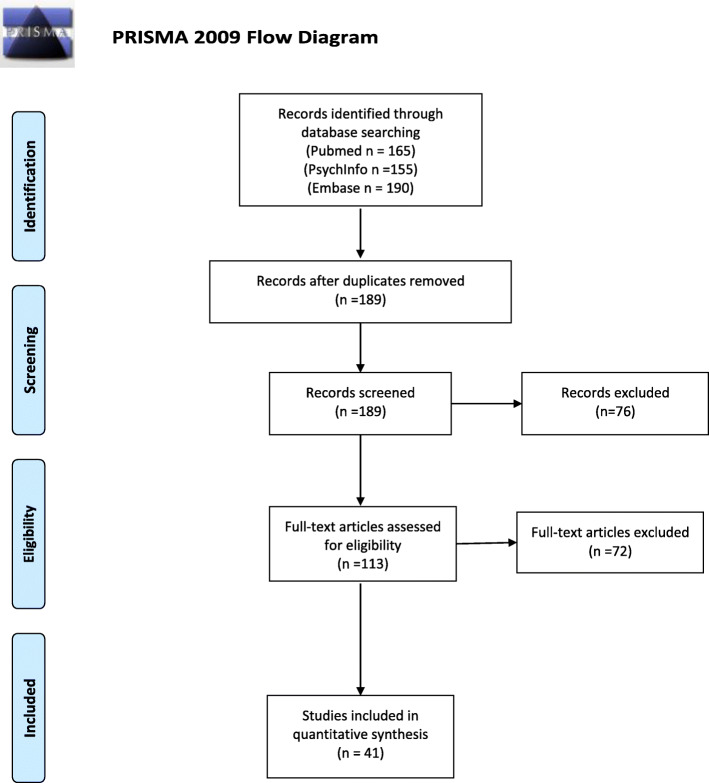
PRISMA 2009 flow diagram

Literature search was performed in PubMed, PsychInfo and Embase databases. A search for [anorexia nervosa] AND [central coherence] resulted in 73 (PubMed), 54 (PsychInfo) and 73 (Embase) articles. A search for [anorexia nervosa] and [set shifting] resulted in 92 (Pubmed), 101 (PsychInfo) and 117 (Embase) articles. See Additional file 1 for search strategy in PubMed. In total, the search resulted in 510 articles. After the initial searches in the different databases, duplicates were removed. Then, all titles and abstracts of 189 articles were examined and evaluated for relevance, which resulted in removal of 76 articles. Then, full text for all potential relevant articles (113), were obtained and screened thoroughly for relevance. Seventy-two articles were then removed due to lack of statistical analyses between neuropsychological function and clinical measures, or they were not relevant for the current review. In all, a total of 41 articles were included in the present study. Figure 1 illustrates a PRISMA flow chart of the search strategy (see Fig. 1).

### Data extraction

The following variables were extracted from the articles: Authors, publication year, sample (size and diagnosis), neuropsychological test, measures of anxiety, depression and eating disorder symptoms, cognitive domain (set-shifting or central coherence), medical treatment. Outcome measures included analyses examining the relationship between set-shifting and/or central coherence and depression/anxiety/BMI/weight for height/eating disorder symptoms.

### Assessment of risk of bias in included studies

Risk of bias of the included studies was assessed by using a critical appraisal checklist for case control studies and cross sectional studies from the Joanna Briggs Institute (https://joannabriggs.org/critical-appraisal-tools), which purpose is to assess the methodological quality of a study and to determine the extent to which a study has addressed the possibility of bias in its design, conduct and analysis. See Additional files 2 and 3.

### Data synthesis

Due to the heterogeneity of the primary studies, especially considering the wide variety of neuropsychological tests and measures of psychopathology, and that different statistical methods have been used, a narrative synthesis approach was used for this current review.

### Study characteristics

Seven studies included an adolescent sample (age < 18 years) [18–24], and four studies included both adults and adolescents [25–28]. The remaining 26 studies included an adult sample (age ≥ 18 years).

A variety of tests were used to measure set-shifting: Haptic Illusion Task, Cognitive Shift Task, Trail Making Test, Brixton Spatial Anticipation Test, Picture Set Test, Verbal Fluency Task, CatBat Task, Intra/Extra Dimensional Set Shift Test, Wisconsin Card Sorting Test, Visual Set-Shifting Task, Berg’s Card Sorting Test, Parametric Go/NoGo Test, Category Learning Task, Task Switching Paradigm, Design Fluency Task, Cued Color-Shape Switching Task and Comprehensive Trail Making Test. The following tests were used to measure central coherence: Rey Complex Figure Test, Embedded Figures Test, Sentence Completion Task, Homograph Reading Task, Overlapping Figures Test, Fragmented Pictures Task, Block Design, Object Assembly and Group Embedded Figures Test.

Different measures were used to assess anxiety and depression. The following measures were used to measure anxiety: State-Trait Anxiety Inventory (*n* = 14), Hospital Anxiety and Depression Scale (*n* = 8), Beck’s Anxiety inventory (*n* = 1), Depression, Anxiety and Stress Scale (*n* = 2), Spence Children’s Anxiety Scale (*n* = 1) and Beck Youth Inventory (*n* = 1). The most common measure of depression was Beck’s Depression Inventory (*n* = 18), then HADS (*n* = 8), Depression, Anxiety and Stress Scale (*n* = 2), Hopkins Symptoms Checklist (*n* = 1), Quick Inventory of Depressive Symptomatology, Children’s Depression Inventory (*n* = 1) and German depression inventory for children and adolescents (*n* = 1).

To examine the associations between cognitive performance and clinical characteristics, different regression analyses accounting for covariates as well as correlation analyses, both Pearson and Spearman’s, were used.

## Results

Table 1 summarises the studies in more detail, including author, sample size (mean age), tests and significant correlations. The studies are organized chronologically according to year of publication. Measure of effect size is not included in the table, as the majority of the studies did not report effect size measures of the association between neuropsychological function and clinical measures.

**Table 1:** Overview of associations between neuropsychological function and clinical measures in patients with AN. Significant associations are highlighted in bold

Authors	Sample ***N*** (mean age)	Test	Anxiety	Depression	BMI/WfH	ED severity
Tchanturia et al., 2002 [29]	AN = 30 (25.2) AN-REC = 16 (30.0) HC = 23 (27.6)	Haptic Illusion Task (SS) Cognitive Shift Task (SS)	Group differences in the Haptic Illusion Task remained after covarying for anxiety**. Group differences in the cognitive shift task disappeared when controlling for anxiety.**	**–**	N/A	N/A
Tchanturia et al., 2004 [30]	AN = 34 (26.7) BN = 19 (26.5) HC = 35 (24.8)	Trail Making Test (SS) Brixton Spatial Anticipation Test (SS) Picture Set Test (SS) Verbal Fluency Task (SS) CatBat Task (SS) Haptic Illusion Task (SS)	**–**	**–**	**–**	N/A
Holliday et al., 2005 [31]	AN = 47 (26.3) AN sisters = 47 (27.6) HC = 47 (26.5)	Haptic Illusion Task (SS) Brixton Spatial Anticipation Test (SS) Trail Making Test (SS) CatBat Task (SS)	**Anxiety scores correlated with the Haptic Illusion Task**.	**Depression scores correlated with the Haptic Illusion Task.**	**–**	N/A
Fowler et al., 2006 [20]	AN = 25 (16.9) HC = 25 (17.6)	Intra/Extra Dimensional Set Shift Test (SS)	**–**	**–**	**–**	N/A
Steinglass et al., 2006 [32, 33]	AN = 15 (25.6) HC = 11 (24.0)	Wisconsin Card Sorting Test (SS)	**–**	**–**	**–**	**–**
Lopez et al., 2008 [5]	AN = 42 (28.4) HC = 42 (26.3)	Rey Complex Figure Test (CC) Embedded Figures Test (CC) Sentence Completion Task (CC) Homograph Reading Task (CC)	**–**	**–**	**BMI correlated with number of errors in the initial pronunciation in the Homograph Reading Task.**	N/A
Kim et al., 2010 [34]	AN = 40 (22.8) BN = 28 (23.0) HC = 34 (22.7)	Trail Making Test (SS)	**–**	**Depression slightly correlated with set-shifting.**	**–**	**–**
Roberts et al., 2010 [35]	AN = 35 (23.7) AN-BP = 33 (25.6) BN = 30 (26.4) AN-REC = 30 (32.1) AN sister = 30 (24.2) BN sister = 20 (27.6) HC = 88 (28.4)	Trail Making Test (SS) Wisconsin Card Sorting Test (SS) Brixton Spatial Anticipation Test (SS) Haptic illusion Task (SS)	**Increased anxiety in the poor set-shifting group, but effects were small**	**Increased depression in the poor set-shifting group, but effects were small.**	**–**	**Those with poor set-shifting had higher score on the YBC-EDS**
Tenconi et al., 2010 [36]	AN = 153 (26.2) HC = 120 (27.4)	Wisconsin Card Sorting Test (SS) Trail Making Test (SS) Rey Complex Figure Test CC) Overlapping Figures Test (CC)	**State anxiety had a moderate correlation with Trail Making Test and Overlapping Figures Test.**	**–**	**–**	**–**
Harrison et al., 2011 [26]	AN = 50 (16–55) BN = 48 (16–55) AN-REC = 35 (16–55) HC = 89 (16–55)	Fragmented Pictures Task (CC) Rey Complex Figure Test (CC) Group Embedded Figures Test (CC)	**–**	**–**	**Negative correlation between BMI and Fragmented Picture Task, but not significant after correcting for multiple comparisons.**	**Those with reduced central coherence had higher EDE-Q Global score**
McAnarney et al., 2011 [27]	AN-R = 24 (14–20) HC = 37 (14–20)	Wisconsin Card Sorting Test (SS) Intra/Extradimensional Set Shift test (SS)	N/A	N/A	No significant correlations.	N/A
Bühren et al., 2012 [37]	AN = 28 (15.6) HC =27 (15.0)	Visual Set-Shifting Task (SS)	**–**	**–**	N/A	N/A
Danner et al., 2012 [38]	AN = 16 (25.6) AN-REC = 15 (24.3) HC = 15 (25.8)	Berg’s Card Sorting Test (SS) Rey Complex Figure Test (CC)	**A better copy accuracy on the Rey Complex Figure Task was related to higher levels of anxiety.**	**–**	N/A	**–**
Galimberti et al., 2012 [39]	AN-R = 24 (26.7) AN-BP = 12 (27.1) BN = 16 (25.3) HC = 40 (25.9)	Intra/Extradimensional Set shift test (SS)	**–**	**–**	**–**	N/A
Giel et al., 2012 [40]	AN = 15 (23.9) UD = 20 (36.3) HC = 35 (30.2)	Trail Making Test (SS) Wisconsin Card Sorting Test (SS) Parametric Go/No-Go Test (SS)	N/A	**Significant medium correlations were found in both AN and UD patients.**	**–**	**–**
Shott et al., 2012 [28]	ADOL-AN = 15 (14.8) ADOL-C = 16 (14.0) ADULT-AN = 26 (26.2) ADULT – C = 33 (26.0)	Category Learning Task (SS)	**–**	**–**	**–**	**–**
Galimberti et al., 2013 [41]	AN = 29 (24.1) HC = 29 (28.6)	Wisconsin Card Sorting Test (SS)	N/A	N/A	**–**	**–**
Tapajoz et al., 2013 [42]	AN = 24 (24.5) BN = 24 (24.4) HC = 24 (25.2)	Rey Complex Figure Task (CC)	**–**	**–**	**–**	**–**
Van Autreve et al., 2013 [43]	AN-R = 31 (26.0) AN-BP = 20 (20.0) HC = 26 (19.0)	Block design (CC) Object Assembly (CC) Wisconsin Card Sorting Test (SS) Trail Making Test (SS) Task Switching Paradigm (SS)	**Trait anxiety in the AN-BP group correlated with Object Assembly.**	**–**	**–**	N/A
Zuchova et al., 2013 [44]	AN = 31 (25.6)	Rey Complex Figure Task (CC)	N/A	N/A	**–**	**Significant correlations between recall accuracy and EDE-Q total score and CR-EAT**
Abbate Daga et al., 2014 [45]	AN = 94 (24.7) HC = 59 (25.1)	Wisconsin Card Sorting Test (SS)	N/A	**AN individuals with poor set-shifting reported higher BDI scores than those with intact set-shifting.**	**–**	N/A
Aloi et al., 2015 [46]	AN-RES = 45 (22.8) BED = 45 (30.6) HC = 45 (25.6)	Rey Complex Figure Test (CC) Trail Making Test (SS) Wisconsin Card sorting Test (SS)	N/A	**–**	**BMI was associated with poor cognitive flexibility, extreme weight conditions performed worse than HC.**	N/A
Lang et al., 2015 [22]	AN = 41 (15.1) HC = 43 (15.1)	Wisconsin Card Sorting Test (SS) Rey Complex Figure Test (CC) Fragmented Pictures Task (CC)	**–**	**–**	**–**	N/A
Renwick et al., 2015 [29]	AN-R = 44 AN-BP = 33 EDNOS-AN = 23 Total *N* = 100 (24.7)	Wisconsin Card Sorting Test (SS) Brixton Spatial Anticipation Test (SS) Rey Complex Figure Test (CC)	N/A	N/A	**–**	N/A
Øverås et al., 2015 [47]	AN-R = 30 (19.1) HC = 45 (18.3)	Wisconsin Card Sorting Test (SS)	**Significant correlations with Wisconsin Card Sorting Test.**	**Significant correlations with Wisconsin Card Sorting Test.**	**Significant correlations with Wisconsin Card Sorting Test.**	N/A
Elzakkers et al., 2016 [48]	AN = 70 (27.3)	Rey Complex Figure Test (CC) Wisconsin Card sorting Test (SS)	N/A	N/A	**–**	N/A
Roberts et al., 2016 [49]	AN = 54 (24.2)	Wisconsin Card Sorting Test (SS) Brixton Spatial Anticipation Test (SS) Trail Making Test (SS) Haptic illusion Task (SS) Rey Complex Figure Test (CC) Group Embedded Figures Test (CC)	N/A	N/A	**The group with both poor set-shifting and global processing had a lower lifetime BMI than patients with one neurocognitive inefficiency.**	N/A
Röβner et al., 2016	AN = 69 (14.3) HC = 63 (14.1)	Trail Making Test (SS)	N/A	**–**	**–**	N/A
Van Noort et al., 2016 [24]	EO-AN =30 (12.2) AN = 30 (15.9) HC = 60 (16.2)	Rey Complex Figure Test (CC) Trail Making Test (SS)	N/A	N/A	**–**	N/A
Van Noort et al., 2016 [24]	AN = 20 (15.6) HC = 20 (15.7)	Trail Making Test (SS) Rey Complex Figure Test (CC)	N/A	N/A	**–**	N/A
Weider et al., 2016 [50]	AN = 41 (28.2) BN = 40 (27.7) HC = 41 (27.7)	Rey Complex Figure Test (CC)	N/A	N/A	**Significant low central coherence index became non-significant when adjusting for nadir BMI.**	N/A
Bentz et al., 2017 [25]	FeAN = 43 (16.1) AN-REC = 28 (18.4) HC = 41 (17.7)	Verbal fluency Task (SS) Design fluency Task (SS) Trail Making Test (SS) Group Embedded Figures Test (CC)	**–**	**–**	N/A	N/A
Perpiña et al., 2016 [51]	AN-R = 18 (22.3) ED.AN = 21 (23.1) BP-G = 47 (24.5) OB = 27 (47.8) HC = 39 (31.9)	Wisconsin Card Sorting Test (SS)	**Positive relationship between anxiety and “total errors” on Wisconsin Card sorting Test. Negative relationship between anxiety and “total categories completed” on the Wisconsin Card Sorting Test.**	**A positive relationship between depression and “total errors” on Wisconsin card sorting test, and negative correlations between depression and Iowa gambling task in all groups.**	N/A	**–**
Øverås et al., 2017 [52]	AN =35 (18.8) HC = 34 (18.7)	Rey Complex Figure Test (CC) Wisconsin Card Sorting Test (SS)	**–**	**–**	**–**	N/A
Brown et al., 2018 [18]	AN = 10 (14.5) HC = 14 (13.7) AN parent =10 (44.7) HC parent =14 (46.9)	Rey Complex Figure Test (CC) Group Embedded Figure Test (CC) Trail Making Test (SS) Verbal Fluency Test (SS)	**Higher levels of anxiety could affect cognitive function.**	**Higher levels of depression could affect cognitive function.**	N/A	N/A
Hamatani et al., 2018 [53]	AN = 22 (31.5) HC = 33 (28.0)	Rey Complex Figure Test (CC)	**–**	**–**	**–**	N/A
Herbrich et al., 2018 [21]	AN-R = 90 (14.4) AN-BP = 21 (15.2) HC = 63 (14.0)	Trail Making Test (SS) Group Embedded Figures Test (CC) Rey Complex Figure Test (CC) Wisconsin Card Sorting Test (SS)	**–**	**–**	**Negative correlation between BMI and Trail Making Test-4 in the AN-R group. Negative correlation between BMI and Group Embedded Figures Test in the AN-BP group.**	N/A
Leppanen et al., 2018 [54]	AN = 145 (25.0)	Rey Complex Figure Test (SS) Brixton Spatial Anticipation Test (SS)	N/A	N/A	**–**	N/A
Oldershaw et al., 2018 [55]	AN = 71 (26.6)	Wisconsin Card Sorting Test (SS) Brixton Spatial Anticipation test (SS) Trail Making Test (SS) Group Embedded Figure Test (CC)	N/A	N/A	**–**	N/A
Berner et al. 2019 [56]	IAN = 40 (24.3) RAN = 24 (25.6) HC = 42 (22.0)	Cued Color-Shape Switching Task (SS)	**–**	**–**	**–**	N/A
Sproch et al. 2019 [57]	AN CRT = 135 (23.9) AN CC = 140 (22.2)	Wisconsin Card Sorting Test (SS) Comprehensive Trail Making Test (SS)	N/A	N/A	**–**	N/A

*ADOL-AN* adolescent patients with AN, *ADOL – C* adolescent controls, *ADULT – AN* adult patients with AN, *ADULT – C* adult control women, *AN* anorexia nervosa, *AN-BP* anorexia nervosa binge/purge subtype, *AN CC* anorexia nervosa control condition, *AN CRT* anorexia nervosa cognitive remediation therapy, *AN-R* anorexia nervosa restrictive subtype, *AN-RES* anorexia nervosa restrictive subtype, *AN-REC* anorexia nervosa recovered, *BDI* Beck’s Depression Inventory, *BED* binge eating disorder, *BN* bulimia nervosa, *CC* Central Coherence, *ED* eating disorder, *ED.AN* eating disorder not otherwise specified anorexia nervosa type, *EO-AN* early onset anorexia nervosa, *FeAN* first-episode AN; HC, healthy controls, *IAN* ill with AN, subthreshold AN or atypical AN, *N/A* not applicable, *BP-G* binge/purge group, *OB* obese group, *RAN* remitted from AN, *SS* set shifting, *UD* patients with unipolar depression, *WfH* weight for height ratio.s

### Risk of bias in included studies

The majority of the studies included in the review had a low risk of bias, i.e. the groups were comparable other than the presence of disease, and the groups were properly matched. Valid, standardized tests were used, and testing procedures were the same for all participants. Confounding variables were identified and controlled for, and appropriate statistical tests were used. However, in some studies, issues concerning representativeness of the patient group and lacking control of possible confounding factors, led to an increased risk of bias. In one study, there was a low participation rate, which could affect the representativeness of the AN group [53]. Another study included a specific sample of AN with strict inclusion criteria, suggesting that this group was not representative for the AN population [55]. One study had a very heterogeneous AN group in terms of differences in duration of illness, age of onset, treatment experience and different levels of illness severity [50]. Furthermore, some of the studies did not control adequately for confounding factors. Most commonly was not considering the effect of pharmacological treatment on test performance [18, 39, 54, 56, 57], and lack of controlling for intelligence quotient [35, 51]. Other potential confounding factors are comorbidity, which was not assessed and controlled for in several studies [44, 56, 57]. These factors contribute to an increased risk of bias in these studies.

### Body mass index and neuropsychological function

The studies that included associations between BMI and neuropsychological function show variable results. The majority of studies (*n* = 28) found no significant associations between set-shifting or central coherence and BMI [20, 22–24, 27–32, 34–36, 39–45, 48, 52–58]. Some studies (*n* = 7) found an association between BMI and neuropsychological function. One study found that BMI correlated with the number of errors in the Homograph Reading Task, a measure of central coherence [5]. A negative correlation was detected between BMI and the Fragmented Picture Task, but the correlation was not significant after correcting for multiple comparisons [26]. Another study found that significant low central coherence index became non-significant when adjusting for nadir BMI [50]. Herbrich et al. [21] found a negative correlation between BMI and Group Embedded Figures Test in the AN-binge/purge group. In addition, a negative correlation was found between BMI and Trail Making Test – 4 in the AN restrictive group. Studies including measures of set-shifting show that BMI was significantly associated with poor cognitive flexibility, were extreme weight conditions performed worse than healthy controls [46]. Øverås et al. demonstrated significant correlations between BMI and Wisconsin Card Sorting Test [47], and Roberts et al. [49] reported that the group with poor set-shifting abilities and global processing had a lower lifetime BMI than patients with only one neurocognitive inefficiency.

### Anxiety and neuropsychological function

Most studies (*n* = 16) did not find an association between neuropsychological function and anxiety [5, 19–22, 25, 26, 28, 30, 32, 34, 39, 42, 52, 53, 56, 59], while nine studies did find a significant association. One study showed that group differences in a Cognitive Shift Task disappeared when controlling for anxiety [59]. Another study found that anxiety scored correlated with the Haptic Illusion task, a measure of set-shifting [31]. Roberts et al. [35] found increased anxiety in the poor set-shifting group, however the effects were small. Another study showed that state anxiety had a moderate correlation with Trail Making Test and Overlapping Figures Test [36]. Øverås et al. and Perpiña et al. also found that set-shifting correlated with anxiety [47, 51]. A better copy accuracy on the Rey Complex Figure Task was related to higher levels of anxiety [38], while trait anxiety in the AN-BP group correlated with Object Assembly, a measure of central coherence. Brown and colleagues [18] suggest that higher levels of anxiety could affect cognitive function.

### Depression and neuropsychological function

A total of 20 studies reported that depression was not associated with neuropsychological function [5, 19–23, 25, 26, 28, 30, 33, 36, 38, 39, 42, 43, 46, 52, 53, 56, 59]. However, nine studies did find significant correlations between depression and measures of set-shifting and central coherence. These studies demonstrate correlations between depression and the Haptic Illusion Task [31], and set-shifting measured by Trail Making Test [34]. Another study found increased depression in the poor set-shifting group, however with small effects [35]. Medium correlations between set-shifting and depression was found in both patients with AN and patients with unipolar depression [40]. Abbate Daga et al. [45] found that AN individuals with poor set-shifting reported higher depression scores, measured by Beck’s Depression Inventory, than those patients intact set-shifting. Furthermore, studies by Øverås et al. [47] and Perpiña et al. [51] show significant correlations between depression and Wisconsin Card Sorting Test. Brown et al. [18] also suggest that higher levels of depression could affect cognitive function such as central coherence and set-shifting.

### Eating disorder severity and neuropsychological function

Some studies investigated the associations between eating disorder severity and neuropsychological functions. Individuals with poor set-shifting had a higher score on the Yale-Brown-Cornell Eating Disorder Scale rituals [35]. Those with most fragmented perseverative cognitive style and the least global flexible style had more chronic and more severe illness, in terms of higher Eating disorder examination questionnaire (EDE-Q) global score and lower BMI [26]. Significant correlations were found between recall accuracy on the Rey Complex Figure Test and total EDE-Q score and scores on the Clinical and Research Inventory for Eating Disorders. In addition, an increase in eating disorder symptoms was related to an improvement in set-shifting measured by the trail making test/TMT [44].

Conversely, other studies showed no correlations between set-shifting performance [32, 40] or central coherence measured by the Rey Complex Figure Task [42] on scores on the Eating Disorder Inventory. Another study found no evidence that symptoms of eating disorders, as measured by EDE-Q, was associated with set-shifting ability [34]. Tenconi et al. [36] divided individuals with AN into three groups, acute AN, weight-recovered but symptomatic AN and long-term fully recovered AN. There were no differences between the groups on measures of set-shifting and central coherence. Furthermore, Danner et al. [38] found no correlations between Iowa Gambling Task and clinical characteristics, while Shott et al. [28] found no significant correlations between shift-cost index on the Category Learning Task and duration of illness. In another study, severity, assessed by the Yale-Brown-Cornell scale scores, BMI, onset and duration of illness, did not correlate with any neurocognitive performance [41]. One study found no significant associations between the Eating Attitudes Test scores and the Iowa Gambling Task total net scores or the Wisconsin Card Sorting Test [51].

## Discussion

The aim of this study was to systematically review the literature to evaluate whether the observed alterations in set-shifting and central coherence are related to BMI or transdiagnostical factors such as anxiety and depression. The JBI Critical Appraisal has shown that the majority studies in this review had a low risk of bias. However, some studies were uncertain of the representativeness of their experimental group, or did not appropriately control for confounding factors such as intelligence quotient, pharmacological treatment or comorbidity, leading to a modest increase in risk of bias in these studies. Main findings, the role of neuropsychological functions in AN and clinical implications will be further discussed.

### Neuropsychological function and clinical measures

The majority of studies report that neither BMI, eating disorder severity, anxiety nor depression is associated with neuropsychological task performance. Deficits in set-shifting and central coherence could thus be related to characteristics of the disorder, and are not a direct consequence of underweight, ED severity, anxiety or depression. However, although most findings points to the same direction, findings are inconsistent. The studies are heterogeneous and methodological differences needs to be considered. Various tests have been used to measure set-shifting and central coherence, one might therefore question whether these results are comparable, as different cognitive tests have different levels of complexity and might target multiple cognitive capabilities. For example, the WCST is primarily a measure of cognitive flexibility, however other cognitive functions such as working memory, inhibition and sustained attention are also necessary to perform this task. The Embedded Figures test appears to the participants to be a measure of attention, but is rather a visuospatial perceptual task measuring central coherence ability. A standardized approach to neuropsychological testing of individuals with AN could contribute to increased consistency across studies, and a better understanding of the neuropathology. Another important issue is that there are numerous measures of psychopathology, and especially for anxiety and depression. Even if they are supposed to measure the same constructs, results from different instruments can be difficult to compare [60].

Furthermore, different statistical methods have been used to determine the relationship between neuropsychological function and clinical measures. Most studies have used correlation analyses (i.e. Pearson or Spearman’s), however some studies did multivariate regression analyses including one or more covariates. The different methods could lead to different conclusions, as a correlation analysis does not adjust for the effect of other covariates.

Neuropsychological performance could also depend on medical treatment. A meta-analysis suggest that deficits in executive functions are greater in patients with more severe current depression, and those taking psychotropic medications [61]. In this review, a few studies accounted for medical treatment and whether it could influence neuropsychological function. Most of these studies suggested that medication did not influence test performance [26, 28, 31, 34, 37], while two studies found differences between medicated and non-medicated patients [36, 42]. Some studies had long-term use of medication [23, 24, 58] and current treatment with psychotropic drugs [25] as exclusion criteria.

It is important to note that cognitive development most likely impact neuropsychological function. Most neuropsychological studies have an adult sample, and studies with an adolescent AN sample are inconsistent, showing both weak and normal set-shifting [27, 62, 63] normal central coherence [21] as well as lower scores on style and central coherence index measured by Rey Complex Figure Test [22]. One study directly compared set-shifting performance in adolescents versus adults with AN and controls [28]. The results showed that set-shifting is normal in adolescents with AN, but impaired in adults, suggesting that neuropsychological difficulties do not precede the disorder, however are rather accompanied by the eating disorder symptoms. Whether neuropsychological function is influenced by clinical measures in adolescents remains unclear. Some studies showed a negative correlation between BMI and both set-shifting and central coherence [21], and that both anxiety and depression could affect cognitive function [18]. Conversely, quite a few studies did not find any significant relationship between set-shifting or central coherence and BMI, anxiety and depression [19, 22–24, 28].

### Neuropsychological function as a link between underlying neurobiological alterations and AN symptoms and behaviour

The main purpose of cognitive neuroscience research in AN is to explain the link between symptoms/behaviour and underlying neural systems. Abnormalities in the fronto-striatal and limbic systems have been suggested to be involved in restrictive eating, which is one of the core symptoms of AN [64]. Central coherence and set-shifting are both executive functions, which rely on frontal lobe activity. More specifically, during a set-shifting task, patients with AN have shown activation in ventrolateral prefrontal cortex, dorsolateral prefrontal cortex, anterior cingulate, superior parietal cortex, cerebellum and precuneus. When performing a central coherence task, parietal, occipital, ventral temporal and premotor cortex is usually activated [65]. Maladaptive behaviours are associated with specific traits, which in turn are associated with neuropsychological processes that are related to specific neural circuits [66]. Clinically, impairments in set-shifting in AN could be linked to excessive control of eating and weight, but also cognitive and behavioural rigidity, which is a common characteristic of the disorder. Patients with AN are often “stuck” in maladaptive behaviours, and have difficulties to change these behavioural patterns. Cognitive and behavioural rigidity could be expressed in terms of being unable to shift perspective from detailed rules and rituals concerning food and eating [6]. Another typical characteristic of individuals with AN, is the increased attention to details, which could be linked to poor global processing. They often have detailed rituals concerning food, caloric intake, exercise and body checking. Taken together, inflexibility and an excessive control of details concerning food and weight, could contribute to the unusual ability to reduce weight and sustain an extreme low weight over a long period.

AN can be very difficult to treat, and there is no proven treatment that can reverse the core symptoms. A complete understanding of neuropsychological functioning in AN, as a link between neural mechanisms and AN symptoms, could contribute to the development of better treatment strategies. As suggested in this review, neuropsychological functions are not influenced by low weight or comorbid disorders, but might be a trait of the illness and should be targeted in treatment with that in mind.

As a result of the emerging neurobiological models of AN, it has been suggested that there is a need for a paradigm shift in the development of new treatment strategies, with more focus on brain-directed treatments [67]. Findings from neuroscientific research has opened up for new avenues for understanding the underlying mechanisms and neuropsychological functions for AN, and for developing new treatments. Temperament based treatment with support (TBT-S) [68] is based on models explaining the underlying neurobiological mechanisms that cause AN. The goal of this treatment is to teach individuals with AN to develop coping strategies for managing their temperament traits that are related to risk factors to develop an eating disorder. The treatment is delivered in a way that is compatible with their neuropsychological thinking style, i.e. detailed oriented, well-structured and predictable. The Maudsley Model of Anorexia Nervosa Treatment for Adults (MANTRA), aims to address the neuropsychological, emotional, relational and biological aspects of AN. It is based on research that has identified the key factors that tend to maintain eating difficulties [69]. The treatment helps patients to understand what causes AN, as well as to encourage patients to change their behaviour when they are ready for it. Cognitive remediation therapy (CRT) aims at improving cognitive flexibility and central coherence, as well as reducing perfectionism and improving the awareness of dysfunctional thinking styles. This treatment is focusing on the process rather than the content of thinking [70].

### Limitations and future directions

Neuropsychological research in AN is distinguished by large varieties of tasks within the same cognitive domain and a variety of measures of psychopathology, which impact and limit interpretation of findings and comparison of results across studies. In a systematic review, a quantitative synthesis is considered to be superior methodologically compared to a narrative synthesis of the results. However, due to the heterogeneity of the primary studies, especially considering the wide variety of neuropsychological tests and measures of psychopathology and that different statistical methods have been used, a narrative synthesis approach was used. Another limitation with the existing literature is that few studies include an adolescent sample, who have a short duration of illness, and addressing this group could contribute to an increased understanding of predisposing factors of AN. Additional issues still needs to be addressed, such as when do the neuropsychological impairments occur? Are neuropsychological impairments related to age or to the progression of the disorder? Do neuropsychological impairments contribute to maintain the disorder? Future studies should target these issues, which could contribute to an increased understanding of the neuropathology of AN.

Future studies would also benefit from controlling for the potential effect of medical treatment on neuropsychological performance, include associations between neuropsychological functioning and eating disorder severity, by including other relevant measures that might have clinical relevance in addition to BMI, e.g. EDE-Q, or other measures of eating disorder symptoms.

## Conclusions

Although the results are inconsistent, the majority of studies in this review report that BMI, anxiety and depression do not influence set-shifting and central coherence capacities in AN. This could indicate that set-shifting and central coherence are characteristic of the disorder per se, and not a consequence of comorbid psychopathologies or emaciation. However, which factors that contribute to altered neuropsychological function in AN are yet to be established. A complete understanding of predisposing, precipitating and maintaining factors of AN needs to be addressed in future research. This could contribute to the development of better and more targeted treatment strategies.

## Supplementary Information

**Additional file 1.** Search strategy in Pubmed. Illustrates the search strategy that was used in PubMed

**Additional file 2.** Checklist_for_Analytical_Cross_Sectional_Studies Critical appraisal tool from the Joanna Briggs Institute, for cross sectional studies included in systematic reviews.

**Additional file 3.** Checklist_for_Case_Control_Studies. Critical appraisal tool from the Joanna Briggs Institute, for case control studies included in systematic reviews.

## Abbreviations

AN: Anorexia nervosa
BMI: Body mass index
CRT: Cognitive remediation therapy
EDE-Q: Eating disorder examination questionnaire
MANTRA: The Maudsley Model of Anorexia Nervosa Treatment for Adults
TBT-S: Temperament based treatment with support
