# Supplementary figures and images for: Is set-shifting and central coherence in anorexia nervosa influenced by body mass index, anxiety or depression? A systematic review
Source: BMC Psychiatry. 2021 Mar 8;21:137. doi: 10.1186/s12888-021-03120-6 (PMC7938561; doi:10.1186/s12888-021-03120-6)

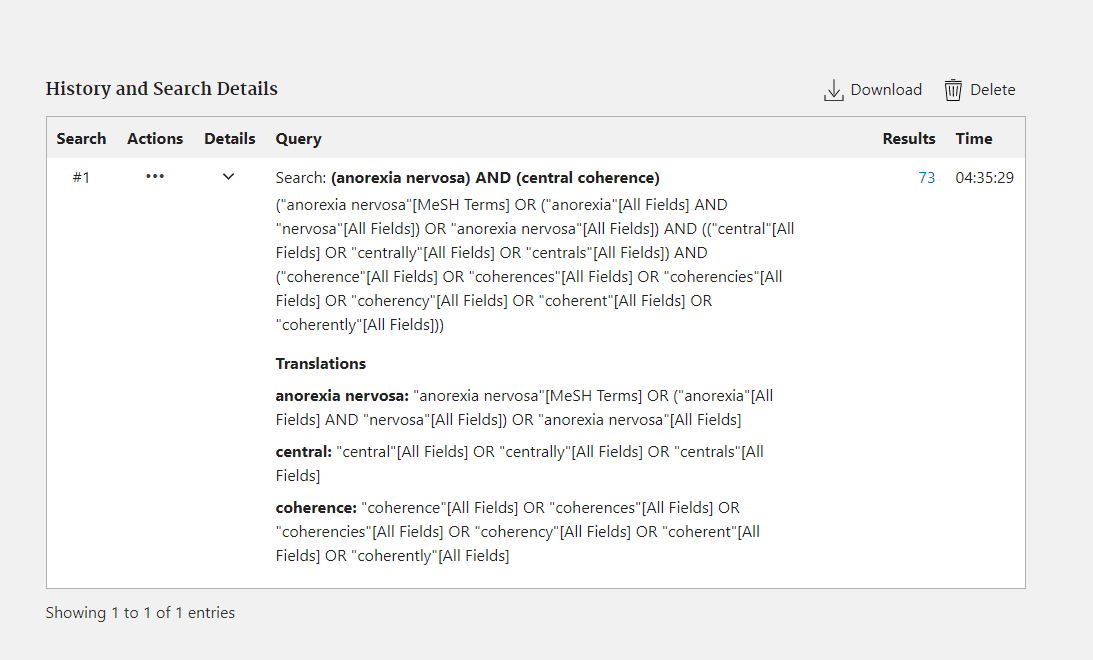

Supplement: Supplementary file 1 — Additional file 1. Search strategy in Pubmed. Illustrates the search strategy that was used in PubMed [file 12888_2021_3120_MOESM1_ESM.png]
